# Analysis of 8 X-chromosomal markers in the population of central Croatia

**DOI:** 10.3325/cmj.2013.54.238

**Published:** 2013-06

**Authors:** Branka Gršković, Anastassiya Zidkova, Vlastimil Stenzl, Maja Popović, Dragan Primorac, Gordan Mršić

**Affiliations:** 1 Forensic Science Centre “Ivan Vučetić,” General Police Directorate, Ministry of Interior, Zagreb, Croatia; 2 University Center for Forensic Sciences, University of Split, Split, Croatia; 3 Institute of Biology and Medical Genetics, First Faculty of Medicine, Charles University in Prague and General University Hospital in Prague, Prague, Czech Republic; 4 Institute of Forensic Medicine and Toxycology, First Faculty of Medicine, Charles University in Prague, Prague, Czech Republic; 5 Department of Forensic Genetics, Institute of Criminalistics, Prague, Czech Republic; 6 Department of Biology, Faculty of Veterinary Medicine, University of Zagreb, Zagreb, Croatia; 7 University of Split, School of Medicine, Split, Croatia; 8 University of Osijek, School of Medicine, Osijek, Croatia; 9 Eberly College of Science, Penn State University, University Park, PA, USA; 10 University of New Haven, New Haven, CT, USA; 11 Genos Ltd, Zagreb, Croatia

## Abstract

**Aim:**

To analyze 8 X-linked short tandem repeat (STR) markers in the population of central Croatia and to evaluate their forensic efficiency.

**Methods:**

We carried out a statistical analysis of the data from previously performed genetic analyses, collected during routine forensic work by the Forensic Science Centre ‘‘Ivan Vučetić.’’ Mentype^®^ Argus X-8 PCR amplification kit was used for typing the data of 99 unrelated healthy women and 78 men from central Croatia. Haplotype frequencies were calculated only in male samples. Arlequin 3.5 software was used to assess Hardy-Weinberg equilibrium (HWE), linkage disequilibrium (LD), observed and expected heterozygosity. Power of discrimination (PD) for men and women, polymorphism information content (PIC), power of exclusion, and mean exclusion chance for deficiency cases, normal trios, and duos were determined using online database ChrX-STR.org.

**Results:**

In female samples, deviations from HWE (*P* = 0.006) for each locus were not found. LD test performed both on female and male samples revealed no significant association between markers (*P* = 0.002). DXS10135 was the most polymorphic locus (PIC = 0.931). PD varied from 0.692 to 0.935 in male and from 0.845 to 0.992 in female samples. Combined PD reached 99.999999% in men and 99.9999999999% in women.

**Conclusion:**

Performed analyses revealed that the studied marker set contained polymorphic markers with high power of discrimination. We can conclude that Mentype^®^ Argus X-8 PCR kit is suitable for application in the population of central Croatia. Results of this study, together with collected allele and haplotype frequencies, are the first step in establishing a national reference X-STR database based on 8 X-STR loci.

The X-chromosome is 155 million base pairs (Mb) long and carries approximately 1250 known genes (1). Men are hemizygous for all X chromosomal markers, while women carry two copies of X chromosome. In the process called X-chromosome inactivation in women, one of their X chromosomes is transcriptionally silenced in a complex and highly coordinated manner according to the Lyon hypothesis (2). A compact structure called Barr body is formed as the inactivated X chromosome condenses. The X chromosome is then stably maintained in a silent state (3). Mutations on the X chromosome occur less frequently than on autosomes due to several times lower nucleotide mutation rate in women than in men, which also reduces the X chromosome genetic diversity (4-7). The X chromosome also shows faster genetic drift than the autosomes as a consequence of the smaller population size (8). This leads to a more pronounced population structure. In women, the overall rate of recombination is higher than in men and it varies along chromosomes in both sexes (9). Recombination occurs extremely rarely around the centromere and at the region Xq13.3-Xq21.3, which is distant from the Xp telomere (10,11). The X chromosome recombines only in women, therefore, a stronger linkage disequilibrium is observed than in autosomes.

Fathers transmit their X chromosome to daughters as haplotypes. X chromosome-linked short tandem repeat (X-STR) loci analysis is used in paternity testing, more complex deficiency paternity cases, when half-sisters and/or grandmothers are to be examined; paternity testing including blood relatives; and rare cases of maternity testing (12). Its advantages are also confirmed in cases when female DNA traces have to be analyzed against a male background. In our study, we used Mentype^®^ Argus X-8, which includes markers that are clustered into 4 linkage groups with 2 closely linked markers per group (DXS10135 and DXS8378; DXS7132 and DXS10074; HPRTB and DXS10101; DXS10134 and DXS7423) (13). Therefore, two markers of each group have to be handled as haplotype for genotyping. Tightly linked STR clusters, which segregate as stable haplotypes, are important for solving complex kinship cases. The usefulness of such clusters is determined by the stability against recombination. It has been already documented that using four tightly linked X-STRs provides stable haplotypes and they have been evaluated for forensic work in several studies (14-19).

To our knowledge, there are no published population data on X-STR diversity in central Croatia. Therefore, the aim of this study was the detailed genetic characterization of 8 X-STR markers in the population of central Croatia. Also, our goal was to define the population structure of this region and to evaluate the forensic efficiency of the used X-STR markers. The association between those 8 X-STR markers was also analyzed. Finally, we performed a population comparison between central Croatian and European and non-European populations.

## Methods

### Study sample

In this study, we followed the guidelines on the use of STRs in forensic analysis created by DNA Commission of the International Society of Forensic Genetics (20-23).

We carried out a statistical analysis of the data from previously performed genetic analyses, collected during routine forensic work by the Forensic Science Centre ‘‘Ivan Vučetić.’’ Data for 99 healthy women and 78 men from the following counties of central Croatia were used: Zagrebačka, Sisačko-moslavačka, Karlovačka, Bjelovarsko-bilogorska, and the city of Zagreb. Participants from all central Croatian counties were included in an attempt to account for any subpopulation variations. The sample size was selected according to the size of the studied population (approximately 2 million) and according to sample sizes in previous studies (16,17,19). The participants were not related and the samples were of sufficient quality and quantity to be included in statistical analysis. The study was approved by the Ethics Committee of the Institute for Medical Research and Occupational Health, Zagreb, Croatia.

### DNA analysis

Genomic DNA from all samples from the materials expertise was extracted from Filter Technology Associates (FTA) cards (Whatman, Maidstone, Kent, UK) and buccal swabs (Whatmann) using Chelex (24). Mentype^®^ Argus X-8 PCR amplification kit (Biotype AG, Dresden, Germany) (13) was used for the amplification of 8 X-STRs: Amelogenin (AM) for sex determination, DXS7132, DXS7423, DXS8378, DXS10074, DXS10101, DXS10134, DXS10135, and HPRTB according to manufacturer's instruction. Mentype® Argus X-8 PCR amplification kit is a highly informative tool for kinship testing, because each of the 4 STR clusters spans less than 0.5 cM and represents a stable haplotype (14,25).

X-STR amplification products were analyzed on 3130xl Genetic Analyzer (Applied Biosystems, Foster City, CA, USA). Data analysis was performed using Genemapper^®^ software (version 3.2, Applied Biosystems). Amplicon sizing was performed using an internal size standard (DNA Size Standard 550 –ROX, Biotype AG), and the amplicons were compared with the Mentype^®^ Argus X-8 allelic ladder (Biotype AG) for unambiguous allele designation.

### Statistical analysis

The allele and haplotype frequencies were determined by counting. Arlequin 3.5 software (26) was used to assess population parameters, perform statistical inference, and compare allele frequencies between different populations. Hardy-Weinberg equilibrium (HWE) exact test, including observed (Ho) and expected heterozygosity (He), was performed for female samples. Linkage disequilibrium (LD) pair-wise loci test was performed both for female and male samples. Haplotype frequencies were calculated only in male samples. For HWE and LD tests, Bonferroni correction was used to determine the significance level. Significance level for HWE testing was 0.006 and for LD testing 0.002. Genetic heterogeneity within population was estimated as gene diversity for male haplotype data. Power of discrimination (PD) for men and women was calculated according to Desmarais et al (27). Other forensic parameters, such as polymorphism information content (PIC), power of exclusion (PE), mean exclusion chance (MEC) for deficiency cases (Krüger’s formula), normal trios (Kishida’s formula) and duos (Desmarais’ formula) were determined using ChrX-STR.org online database that calculates population-genetic data (28). The haplotype frequencies from central Croatia were compared with those from German, Japanese, and Ghanaian population samples by applying exact test of population differentiation. For comparison with Polish population, exact test based on allele frequencies was used, because no haplotype frequencies were available in ChrX-STR.org online database (28). Exact test based on allele frequencies was also used for comparison with the population of Bosnia and Herzegovina for the loci DXS8378, DXS7132, HPRTB, and DXS7423. Arlequin 3.5 software was used for allele and haplotype frequencies comparison. Significance level for interpopulation comparison was set to 0.025 after Bonferroni correction (26).

## Results

We determined allele frequencies, Ho and He, and *P* values for the HWE of 8 X-STR in the population of central Croatia (Table 1). Seven variant alleles were observed at the DXS10135 (15.3, 19.1, 20.1, 21.1, 22.1, 23.1, 25.1), 1 at the HPRTB (11.2), 10 at the DXS10101 (25.2, 26.2, 27.2, 28.2, 29.2, 30.2, 31.2, 32.2, 33.2, and 34.2), and 11 at the DXS10134 (30.2, 35.2, 37.1, 37.2, 37.3, 38.3, 39.3, 40.3, 41.3, 42.3, and 43.3). The variant alleles 15.3 at the DXS10135 and 30.2 and 37.1 at the DXS10134 locus were found for the first time in the population of central Croatia. Genotype proportions in female samples for each locus did not show deviations from HWE (Table 1).

**Table 1:** Allele frequencies at 8 X-short tandem repeat loci in the population of central Croatia (N = 177)*

Allele	DXS10135	DXS8378	DXS7132	DXS10074	HPRTB	DXS10101	DXS10134	DXS7423	Allele
7				0.03986	0.02536				7
8				0.15217					8
9		0.01449		0.00725					9
10		0.32971			0.01087				10
11		0.36957	0.00362	0.00362	0.10870				11
11.2					0.00362				11.2
12		0.24638	0.10870	0.00362	0.31884				12
13		0.03986	0.32609	0.01087	0.30797			0.09783	13
14			0.33333	0.01087	0.19203			0.31884	14
15			0.17391	0.06884	0.02536			0.41667	15
15.3	0.00362								15.3
16	0.00362		0.04348	0.22464	0.00725			0.13406	16
17	0.01087		0.01087	0.24638				0.03261	17
18	0.04348			0.16667					18
19	0.06159			0.05797					19
19.1	0.00362								19.1
20	0.04348			0.00725					20
20.1	0.03261								20.1
21	0.06159								21
21.1	0.02174								21.1
22	0.05797								22
22.1	0.00725								22.1
23	0.08333								23
23.1	0.01449								23.1
24	0.08696								24
25	0.09420								25
25.1	0.00725								25.1
25.2						0.01449			25.2
26	0.09420								26
26.2						0.00362			26.2
27	0.09783					0.00362			27
27.2						0.05072			27.2
28	0.05072					0.02174			28
28.2						0.12319			28.2
29	0.04710					0.06884			29
29.2						0.12319			29.2
30	0.03986					0.02899	0.00362		30
30.2						0.16304	0.00362		30.2
31	0.02174					0.08333	0.01087		31
31.2						0.11232			31.2
32	0.00725					0.07609	0.00362		32
32.2						0.04710			32.2
33						0.04710	0.05435		33
33.2						0.01087			33.2
34	0.00362					0.01812	0.12681		34
34.2						0.00362			34.2
35							0.19928		35
35.2							0.00362		35.2
36							0.20652		36
37							0.17029		37
37.1							0.00362		37.1
37.2							0.00725		37.2
37.3							0.00725		37.3
38							0.06159		38
38.3							0.02536		38.3
39							0.01087		39
39.3							0.03986		39.3
40							0.00362		40
40.3							0.02174		40.3
41							0.00362		41
41.3							0.01449		41.3
42.3							0.01087		42.3
43.3							0.00725		43.3
*P*	0.807	0.249	0.498	0.082	0.730	0.347	0.6646	0.362	*P*
SD	0.000	0.000	0.000	0.000	0.000	0.000	0.000	0.000	SD
Ho	0.95960	0.67677	0.75758	0.80808	0.78788	0.89899	0.84848	0.76768	Ho
He	0.94011	0.69656	0.74348	0.82900	0.76050	0.89781	0.85992	0.70164	He

*Abbreviations: *P*-value – result of Hardy-Weinberg equilibrium test with a significance level 0.006; SD – standard deviation; Ho – observed heterozygosity; He – expected heterozygosity.

All *P*-values obtained by HWE test were higher than the significance level that was 0.006 after Bonferroni correction. LD test, which was performed both on female and male samples, revealed no significant association between the studied markers, because all *P*-values were higher than the significance level after applying Bonferroni correction (0.002) (Table 2). Although LD test did not found a significant linkage between studied markers, this could be explained by the small sample size.

**Table 2:** Linkage disequilibrium (LD) for 8 X-linked markers in four linkage groups*

Marker pair	Linkage group	LD test female		LD test male	
		*P*	SD	*P*	SD
DXS10135/DXS8378	1/1	0.122	0.003	0.010	0.001
DXS10135/DXS7132	1/2	0.661	0.005	0.296	0.001
DXS10135/DXS10074	1/2	0.520	0.005	0.302	0.002
DXS10135/HPRTB	1/3	0.919	0.003	0.437	0.003
DXS10135/DXS10101	1/3	0.070	0.003	0.115	0.001
DXS10135/DXS10134	1/4	0.147	0.004	0.480	0.002
DXS10135/DXS7423	1/4	0.185	0.004	0.221	0.002
DXS8378/DXS7132	1/2	0.445	0.005	0.518	0.004
DXS8378/DXS10074	1/2	0.527	0.005	0.915	0.002
DXS8378/HPRTB	1/3	0.397	0.005	0.819	0.002
DXS8378/DXS10101	1/3	0.286	0.005	0.101	0.002
DXS8378/DXS10134	1/4	0.587	0.006	0.873	0.002
DXS8378/DXS7423	1/4	0.310	0.005	0.556	0.003
DXS7132/DXS10074	2/2	0.677	0.005	0.545	0.003
DXS7132/HPRTB	2/3	0.788	0.004	0.877	0.001
DXS7132/DXS10101	2/3	0.758	0.004	0.449	0.002
DXS7132/DXS10134	2/4	0.761	0.004	0.293	0.001
DXS7132/DXS7423	2/4	0.767	0.004	0.765	0.003
DXS10074/HPRTB	2/3	0.482	0.005	0.931	0.002
DXS10074/DXS10101	2/3	0.869	0.004	0.270	0.002
DXS10074/DXS10134	2/4	0.031	0.002	0.522	0.002
DXS10074/DXS7423	2/4	0.210	0.004	0.140	0.002
HPRTB/DXS10101	3/3	0.003	0.000	0.054	0.001
HPRTB/DXS10134	3/4	0.007	0.000	0.569	0.001
HPRTB/DXS7423	3/4	0.808	0.004	0.182	0.002
DXS10101/DXS10134	3/4	0.856	0.004	0.763	0.001
DXS10101/DXS7423	3/4	0.263	0.004	0.522	0.003
DXS10134/DXS7423	4/4	0.872	0.003	0.021	0.001

*Abbreviations: *P*-value – result of linkage equilibrium test with a significance level 0.002; SD – standard deviation, abbreviations for loci in the linkage groups: 1. DXS10135 and DXS8378; 2. DXS7132 and DXS10074; 3. HPRTB and DXS10101; 4. DXS10134 and DXS7423.

We compared the allele frequencies from our study to those from the population of southern Poland (29), which is also Slavic in origin. Also, we compared them to the closely located population of Bosnia and Herzegovina (30). No significant differences were found for both populations; significance level was 0.025 after Bonferroni correction (Table 3).

**Table 3:** Allele frequencies comparison of the population from central Croatia and populations from Poland and Bosnia and Herzegovina*

	Polish population		Bosnian and Herzegovinian population	
Locus	*P*	SD	*P*	SD
DXS10135	0.159	0.014	NA	NA
DXS8378	0.142	0.008	0.667	0.059
DXS7132	0.215	0.013	0.441	0.063
DXS10074	0.972	0.002	NA	NA
HPRTB	0.293	0.019	0.324	0.051
DXS10101	0.056	0.008	NA	NA
DXS10134	0.546	0.025	NA	NA
DXS7423	0.800	0.008	0.657	0.039

*SD – standard deviation, NA – allele frequencies data were not available.

Haplotype frequencies of four linkage groups (LG) were counted in 78 men. The linkage groups 1, 2, 3, and 4 revealed 37, 30, 35 and 30 haplotypes, respectively (Table 4). Moreover, gene diversity in central Croatian population was higher than 0.95 for each LG (Table 4). The most frequent haplotypes were 25-12 and 24-12 for LG1; 13-16 for LG2; 13-31.2 for LG3; and 35-15 for LG4. Haplotype frequencies for each LG were compared with haplotype frequencies from Japan, Ghana, and Germany (14) (Table 5). Significant differences were found for every LG for Japanese and Ghanaian population, *P*-values were lower than the significance level (0.025 after applying Bonferroni correction).

**Table 4:** Haplotype frequencies for 8 X-linked markers in four linkage groups in central Croatia (N = 78 men)*

Linkage group 1				Linkage group 2				Linkage group 3				Linkage group 4			
DXS10135 - DXS8378		N	Frequency	DXS7132 - DXS10074		N	Frequency	HPRTB - DXS10101		N	Frequency	DXS10134 - DXS7423		N	Frequency
17	12	1	0.0128	11	16	1	0.0128	9	30.2	2	0.0256	31	15	1	0.0128
18	10	2	0.0256	12	15	1	0.0128	10	30.2	1	0.0128	32	17	1	0.0128
18	12	2	0.0256	12	17	2	0.0256	11	28.2	1	0.0128	33	15	3	0.0385
19	10	2	0.0256	12	18	4	0.0513	11	29.2	4	0.0513	33	16	1	0.0128
19	11	1	0.0128	12	19	1	0.0128	11	30	2	0.0256	33	17	1	0.0128
19	12	3	0.0385	13	8	4	0.0513	11	30.2	1	0.0128	34	14	3	0.0385
20.1	10	3	0.0385	13	15	2	0.0256	11	31.2	2	0.0256	34	15	3	0.0385
20.1	12	1	0.0128	13	16	9	0.1154	11	32.2	1	0.0128	34	16	6	0.0769
21	10	4	0.0513	13	17	5	0.0641	12	25.2	1	0.0128	34	17	1	0.0128
21	12	1	0.0128	13	18	3	0.0385	12	27.2	2	0.0256	35	14	5	0.0641
21.1	10	2	0.0256	13	19	2	0.0256	12	28.2	2	0.0256	35	15	8	0.1026
22	10	1	0.0128	14	7	4	0.0513	12	29	1	0.0128	35	16	3	0.0385
22	11	1	0.0128	14	8	4	0.0513	12	29.2	5	0.0641	36	14	1	0.0128
22	12	1	0.0128	14	13	2	0.0256	12	30.2	4	0.0513	36	13	6	0.0769
23	10	2	0.0256	14	15	3	0.0385	12	31	2	0.0256	36	15	6	0.0769
23	11	2	0.0256	14	16	4	0.0513	12	32	1	0.0128	37	13	1	0.0128
23	12	2	0.0256	14	17	6	0.0769	12	32.2	3	0.0385	37	14	5	0.0641
23.1	10	1	0.0128	14	18	3	0.0385	12	34	1	0.0128	37	15	4	0.0513
23.1	11	1	0.0128	14	19	2	0.0256	13	28.2	4	0.0513	37.1	15	1	0.0128
24	10	1	0.0128	14	20	1	0.0128	13	29.2	5	0.0641	37.3	15	1	0.0128
24	11	3	0.0385	15	8	1	0.0128	13	30.2	1	0.0128	38	13	1	0.0128
24	12	6	0.0769	15	14	1	0.0128	13	31	1	0.0128	38	14	2	0.0256
25	10	4	0.0513	15	16	1	0.0128	13	31.2	8	0.1026	38	15	2	0.0256
25	11	1	0.0128	15	17	4	0.0513	13	32	4	0.0513	38.3	14	1	0.0128
25	12	6	0.0769	15	18	2	0.0256	13	32.2	2	0.0256	38.3	16	2	0.0256
26	11	5	0.0641	15	19	1	0.0128	13	33	5	0.0641	39	13	1	0.0128
26	12	1	0.0128	16	8	1	0.0128	14	28	1	0.0128	39.3	14	1	0.0128
27	10	2	0.0256	16	17	2	0.0256	14	29	1	0.0128	39.3	15	5	0.0641
27	11	3	0.0385	17	8	1	0.0128	14	29.2	3	0.0385	40.3	16	1	0.0128
27	12	1	0.0128	17	17	1	0.0128	14	30	1	0.0128	42.3	17	1	0.0128
28	10	2	0.0256					14	30.2	2	0.0256				
28	11	1	0.0128					14	31	1	0.0128				
28	12	1	0.0128					14	31.2	1	0.0128				
28	13	1	0.0128					14	32	1	0.0128				
29	11	2	0.0256					15	31	1	0.0128				
30	11	4	0.0513												
31	11	1	0.0128												
Gene diversity	0.9734	SD	0.0061	Gene diversity	0.9624	SD	0.0078	Gene diversity	0.9680	SD	0.0071	Gene diversity	0.9587	SD	0.0076

*SD – standard deviation.

**Table 5:** Haplotype frequencies comparison of the population from central Croatia and populations from Japan, Ghana, and Germany*

Linkage group	*P* (Croatian vs Japanese)	SD	*P* (Croatian vs Ghanaian)	SD	*P* (Croatian vs German)	SD
1	0.008	0.003	0.003	0.002	0.115	0.052
2	<0.001	0.000	<0.001	0.000	0.385	0.059
3	<0.001	0.000	<0.001	0.000	0.053	0.022
4	<0.001	0.000	0.008	0.003	0.003	0.003

*SD – standard deviation.

DXS10135 was the most polymorphic locus (with 25 alleles, PIC = 0.9306), whereas the lowest values were observed for DXS7423 and DXS8378 (both with 5 alleles, PIC 0.6316 and 0.6447, respectively). PIC for the whole marker set was 0.999998. PD ranged from 0.6922 to 0.9345 in male samples and from 0.8447 to 0.9918 in female samples. Combined PD reached 99.999999% in men and 99.9999999999% in women (Table 6).

**Table 6:** Forensic parameters for 8 X-linked markers in the population of central Croatia*

Locus	Number of alleles	PD_men_	PD_women_	PIC	PE	MEC_Krüger_	MEC_Kishida_	MEC_Desmarais Duo_
DXS10135	25	0.9345	0.9918	0.9306	0.8663	0.8670	0.9304	0.8741
DXS8378	5	0.6922	0.8447	0.6316	0.4163	0.4230	0.6316	0.4857
DXS7132	7	0.7385	0.8879	0.6948	0.4903	0.5055	0.6948	0.5553
DXS10074	14	0.8278	0.9483	0.8058	0.6517	0.6596	0.8058	0.6907
HPRTB	10	0.7533	0.8992	0.7133	0.5155	0.5295	0.7133	0.5767
DXS10101	19	0.9040	0.9830	0.8962	0.8036	0.8061	0.8960	0.8192
DXS10134	23	0.8623	0.9666	0.8479	0.7192	0.7271	0.8476	0.7488
DXS7423	5	0.6961	0.8562	0.6447	0.4223	0.4483	0.6447	0.5004
Combined		0.999999	0.9999999999	0.999998	0.99978	0.99982	0.999998	0.999914

*Abbreviations: PD – power of discrimination; PIC – polymorphism information content; PE – power of exclusion; MEC – mean exclusion chance for deficiency cases, normal trios, and duos. For forensic parameters calculation, total allele frequencies were used.

## Discussion

We found variant alleles 15.3 at the DXS10135 and 30.2 and 37.1 at the DXS10134 loci, which is the first time these alleles were found in the population of central Croatia using Mentype® Argus X-8 PCR amplification kit. A previous study on the population from Bosnia and Herzegovina (30) investigated 4 mini X-STRs, divided into 7 pairs of markers (GATA172D05-GATA31E08, DXS7132-DXS7423, DXS9902-HPRTB, DXS7130-DXS6803, DXS6789-GATA172D05, DXS8378-GATA172D05, and DXS7424-DXS130) but since they did not use Mentype® Argus X-8 kit, we could compare only four common loci, DXS8378, DXS7132, HPRTB, and DXS7423. It is interesting that allele 8 at the HPRTB locus was found only in the population from Bosnia and Herzegovina and allele 11.2 was found only in central Croatia.

Genetic heterogeneity within the population of central Croatia was very high for each LG. Gene diversity for female (equivalent to expected heterozygosity for diploid data) and male data in the population was relatively high and close to 1 for the most informative markers, DXS10135 and DXS10101. The fact that no gametic association was proven between the loci, both in female and male samples, could be explained by high mutation rates for X-STR, which has already been observed in other populations (14,20). Obtained haplotype frequencies should be taken into account when a set of more than a single meiosis is considered (21).

Significant differences in all LGs between central Croatia, Japan, and Ghana could be explained by a relatively large genetic distance between these populations. Although the population of central Croatia is genetically closer to German population, significant difference in haplotype frequencies was found in LG4. This could be due to large differences in the sample size between our (N = 78 men) and German population (N = 439 men) (14).

Forensic parameters for 8 X-STR markers in central Croatian population were comparable to those published elsewhere (14-20,22,29). DXS10135, DXS10101, and DXS10134 with the PIC value close to 1 (0.9306, 0.8962 and 0.8478, respectively) increased the discrimination power of 8 X-STRs in central Croatia. Other forensic parameters, such as PD, PE, and MEC indicate that Argus X-8 PCR amplification kit is suitable for forensic and kinship analysis in the population of central Croatia, in cases when autosomal STR markers do not provide the needed information. The X-STR data for central Croatia obtained in this study were submitted to ChrX-STR.org online database (28).

In a complex kinship testing, X-STR genotyping can supplement the analysis of autosomal, mitochondrial, and Y-chromosomal markers. In the last few years, the need for commercially available and validated X-STR kits has increased due to a growing number of complex kinship cases. Intensive studies of the X chromosome discover a lot of closely linked X-STR markers, which can be included in the commercially available kits (31-35).

Recent forensic casework, population genetics, and anthropological studies have used the relatively new commercially available Investigator Argus X-12 kit (36). Investigator Argus X-12 amplification kit presents an improvement compared to Mentype^®^ Argus X-8 in the sense of increased discriminatory power due to four linkage groups with three markers per group (Amelogenin; DXS10148, DXS10135, DXS8378; DXS7132, DXS10079, DXS10074; DXS10103, HPRTB, DXS10101; DXS10146, DXS10134, DXS7423) (36). In addition, in genotyping three markers of each group should be handled as a haplotype. The kit was successfully validated by Edelmann et al (37). More and more X-STR markers are appearing on the forensic scene. Even though the number of cases that require X-STRs analysis is still quite small, forensic community needs to have an appropriate genetic tool to solve any complex kinship case.

To the best of our knowledge, this is the first population study of 8 X-linked STRs in Croatia. All the analyzed markers were in HWE; therefore Hardy-Weinberg laws could be applied for match probability calculation. The limitation of this study is the relatively small sample size, which could be further extended to test LD.

X-linked markers in Mentype® Argus X-8 PCR amplification kit proved to be highly polymorphic with a high power of discrimination. Mentype® Argus X-8 was shown to be a robust kit that could be used as an additional marker panel for forensic identification, paternity testing, and kinship determination. The collected allele and haplotype frequencies data could help to establish X-STR kinship and identification analysis in central Croatia. The results of our study will be included in the Croatian national reference X-STR database based on 8 loci. Further studies are planned to get an overview of the X-STR variability in all Croatian regions, and there are plans for inclusion of 12 X-STR loci in the database. The implementation of additional marker set included in Investigator Argus X-12 kit would increase discriminatory power as a very important prerequisite for further enlargement of Croatian national reference X-STR database.
